# Biomarkers for Clinical Benefit of Immune Checkpoint Inhibitor Treatment—A Review From the Melanoma Perspective and Beyond

**DOI:** 10.3389/fimmu.2018.01474

**Published:** 2018-06-28

**Authors:** Kristina Buder-Bakhaya, Jessica C. Hassel

**Affiliations:** Section of Dermatooncology, Department of Dermatology and National Center for Tumor Diseases (NCT), University Hospital Heidelberg, Heidelberg, Germany

**Keywords:** biomarker, checkpoint inhibition, PD-1 antibody, PD-L1 antibody, CTLA-4 antibody, cancer, melanoma

## Abstract

**Background:**

Immune checkpoint inhibition (ICI) with anti-CTLA-4 and/or anti-PD-1 antibodies is standard treatment for metastatic melanoma. Anti-PD-1 (pembrolizumab, nivolumab) and anti-PD-L1 antibodies (atezolizumab, durvalumab, and avelumab) have been approved for treatment of several other advanced malignancies, including non-small-cell lung cancer (NSCLC); renal cell, and urothelial carcinoma; head and neck cancer; gastric, hepatocellular, and Merkel-cell carcinoma; and classical Hodgkin lymphoma. In some of these malignancies approval was based on the detection of biomarkers such as PD-L1 expression or high microsatellite instability.

**Methods:**

We review the current status of prognostic and predictive biomarkers used in ICI for melanoma and other malignancies. We include clinical, tissue, blood, and stool biomarkers, as well as imaging biomarkers.

**Results:**

Several biomarkers have been studied in ICI for metastatic melanoma. In clinical practice, pre-treatment tumor burden measured by means of imaging and serum lactate dehydrogenase level is already being used to estimate the likelihood of effective ICI treatment. In peripheral blood, the number of different immune cell types, such as lymphocytes, neutrophils, and eosinophils, as well as different soluble factors, have been correlated with clinical outcome. For intra-tumoral biomarkers, expression of the PD-1 ligand PD-L1 has been found to be of some predictive value for anti-PD-1-directed therapy for NSCLC and melanoma. A high mutational load, particularly when accompanied by neoantigens, seems to facilitate immune response and correlates with patient survival for all entities treated by use of ICI. Tumor microenvironment also seems to be of major importance. Interestingly, even the gut microbiome has been found to correlate with response to ICI, most likely through immuno-stimulatory effects of distinct bacteria. New imaging biomarkers, e.g., for PET, and magnetic resonance imaging are also being investigated, and results suggest they will make early prediction of patient response possible.

**Conclusion:**

Several promising results are available regarding possible biomarkers for response to ICI, which need to be validated in large clinical trials. A better understanding of how ICI works will enable the development of biomarkers that can predict the response of individual patients.

## Introduction

In the last decade, treatment of metastatic melanoma and other malignancies has improved significantly. In addition to targeted treatment options, immunotherapy with immune checkpoint inhibitors (ICI) has contributed greatly to this development.

The anti-CTLA-4 antibody (CTLA4ab) ipilimumab was first approved by the U.S. Food and Drug Administration (FDA) for the treatment of metastatic melanoma in 2011 (1), followed by the anti-PD-1 antibodies (PD1ab) pembrolizumab and nivolumab in 2014 (2–4). Combined ICI with CTLA4ab and PD1ab for melanoma was introduced with enormous success, but was also accompanied by significant immune-related adverse events (irAEs) (5, 6). PD1ab treatment is currently approved for treatment of several other advanced malignancies including non-small-cell lung cancer (NSCLC), urothelial cancer, renal cell carcinoma (RCC), squamous cell carcinoma of head and neck (SCCHN), gastric carcinoma, hepatocellular carcinoma, and classical Hodgkin lymphoma (7–12). The anti-PD-L1 antibodies (PD-L1ab) atezolizumab (urothelial carcinoma and NSCLC), durvalumab (urothelial carcinoma), and avelumab [Merkel-cell carcinoma (MCC) and urothelial carcinoma] have also recently been approved by the FDA (13–18). In 2017 the FDA also announced a biomarker-based approval for pembrolizumab for patients with unresectable or metastatic solid tumors, and for colorectal carcinoma (CRC) with high microsatellite instability or mismatch repair deficiency (dMMR) (19).

Despite this enormous success, ICI does not achieve long-lasting responses for all patients. Response varies between different entities, and between different patients. For melanoma, PD1ab monotherapy can achieve a response of 26–32% (2, 4) and the combination of PD1ab and CTLA4ab achieves a response as high as 60% (5). Some subsets of patients achieve durable responses with PD1ab monotherapy and do not require combined ICI, and could therefore be protected from the higher risk of irAEs.

There remains the medical need to find reliable biomarkers that could help to identify both, the patients who would benefit from ICI and the primary resistant patients. Biomarkers are also needed to help decide the type of first-line treatment, e.g., whether BRAF-mutant melanoma should be treated by use of targeted or immunotherapy, therapy sequencing, and/or by a combination of treatments. Here, we review biomarkers in the field of ICI therapy for metastatic melanoma and other malignancies. We have not performed a review of pre-analytic, analytic, and clinical validation techniques for biomarkers because these have been reviewed elsewhere (20, 21).

## Clinical Biomarkers

### Tumor Burden

Tumor burden and metastatic site, e.g., liver or brain metastases, significantly affect patient prognosis, particularly in terms of overall survival (OS), as described in the TNM classification (22). Because the prognostic effect of tumor burden and metastatic site is well known, they are used to stratify clinical trials and are the object of sub-group analyses. Several authors have found an association between metastatic site and incidence of response, progression-free survival (PFS), and OS for PD1ab treatment of melanoma (23–26) (Table 1). Response to PD1ab therapy is better for lung and skin metastases than for metastases in other organs, particularly those in the liver. Response for melanoma brain metastases is lower compared with response for extracranial sites, particularly for PD-1ab monotherapy (27). This could be because T cell infiltrate in cerebral metastases is less dense compared with other anatomic sites (28). For combined ICI with ipilimumab and nivolumab, response for brain metastases that were asymptomatic was similar to response for extracranial sites (27, 29). Peripheral blood biomarkers which correlate with tumor burden, such as serum lactate dehydrogenase (LDH), circulating tumor cells (CTCs), and circulating tumor DNA (ctDNA), are of significance for biomarker investigations, as described below.

**Table 1:** Clinical, blood, and stool biomarkers for clinical outcome under checkpoint blockage for metastatic melanoma patients.

Biomarker	Number of patients	Treatment	Results	Reference
**Clinical Biomarkers**				

Metastatic sites	*n* = 177–593	Pembrolizumab, nivolumab	Liver metastases associated with lower response rate and shorter PFS	(26) (24) (25)

	*n* = 177–257	Pembrolizumab	Soft-tissue and/or lung metastases associated with longer OS	(23)

ECOG performance status	*n* = 50	Nivolumab	ECOG PS ≥ 1 associated with shorter OS	(30)

Immune-related adverse events (irAEs)	*n* = 86	Ipilimumab, nivolumab	irAEs associated with response	(31) (32)

	*n* = 154	Ipilimumab	Hypophysitis associated with longer OS	(33)

	*n* = 65–118	Pembrolizumab, nivolumab	Vitiligo associated with response and longer OS	(34) (35)

	*n* = 196	Pembrolizumab, nivolumab	Arthralgia associated with response and longer OS	(36)

	*n* = 298–833	Ipilimumab	No association of irAEs with response or survival	(37) (38)

**Blood Biomarkers**				

LDH	*n* = 50–257	Ipilimumab, nivolumab, pembrolizumab, ipilimumab + nivolumab, or pembrolizumab	Elevated LDH associated with shorter OS	(39) (40) (41) (31) (30) (42) (23) (43) (44)

CRP	*n* = 95–196	Ipilimumab	CRP within normal limits associated with longer OS	(43) (45)

	*n* = 50	Nivolumab	CRP not significant for OS in multivariable analysis	(30)

Neutrophils	*n* = 50–720	Ipilimumab	Elevated neutrophils associated with shorter OS	(40) (30) (46)

Lymphocytes	*n* = 50–257	Ipilimumab, pembrolizumab, nivolumab	Absolute lymphocyte counts (LC) ≥1,000/ml, high relative LC or increasing LC with treatment associated with longer OS	(45) (23) (30) (41, 47)

NLR	*n* = 58–720	Ipilimumab, nivolumab	Baseline NLR ≥ 3–4 associated with shorter OS	(46) (48) (49)

	*n* = 90	Nivolumab	Baseline NLR ≥ 2.2 associated with non-response	(50)

Eosinophils	*n* = 59	Ipilimumab	Increase in eosinophil count (week 3) associated with response	(51)

	*n* = 177–209	Ipilimumab, pembrolizumab	High eosinophils associated with longer OS	(41) (23)

Monocytes, mo-MDSCs	*n* = 20–209	Ipilimumab, nivolumab, pembrolizumab	Elevated mo-MDSCs/monocytes associated with non-response and shorter PFS/OS	(51) (41) (52) (49) (53)

T cell subsets	*n* = 95–209	Ipilimumab	High Treg count associated with longer OS	(45) (41)

	*n* = 67–82	Ipilimumab	Relative numbers of CD4^+^ and CD8^+^ T cells correlated with response and longer OS	(47) (54)

	*n* = 37–190	Ipilimumab	Higher PD-L1 expression on peripheral T cells correlated with non-response, shorter PFS and OS	(55)

	*n* = 67	Nivolumab, pembrolizumab	NK cell subsets associated with response to PD1ab	(54)

Human leukocyte antigen class I genotype (HLA-I)	*n* = 1535 (mainly melanoma, NSCLC)	Ipilimumab ± nivolumab, nivolumab, pembrolizumab	Maximum heterozygosity at HLA-I loci correlated with longer OS, melanoma only: HLA-B44 supertype associated with longer OS, HLA-B62, or somatic loss of heterozygosity at HLA-I associated with shorter OS	(56)

T cell receptor (TCR) repertoire	*n* = 12	Ipilimumab	TCR repertoire richness prior to therapy correlated with clinical benefit	(57)

sCTLA4	*n* = 14	Ipilimumab	sCTLA4 higher in responders, associated with longer OS	(58)

sPD-L1	*n* = 251	Ipilimumab (±bevazizumab or sargramostim), pembrolizumab	High pretreatment levels associated with disease progression	(59)

sULBP-1, sULBP-2	*n* = 194 (ICI) *n* = 65 (other treatments)	Ipilimumab ± nivolumab or pembrolizumab, nivolumab	sULBP-1 and 2 associated with disease control and longer OS in ICI, but not in other treatments	(44)

sCD25	*n* = 27	Ipilimumab	High baseline sCD25 associated with shorter OS	(60)

CXCL11	*n* = 48–247	Ipilimumab or gp100 peptide vaccine	Pre-treatment elevated serum CXCL11 level associated with shorter OS	(61)

Cytokine levels	*n* = 35	Nivolumab	Serum IFN-γ, IL-6, and IL-10 levels higher for responders	(62)

Protein signature (multimarker assay)	*n* = 119–170	Ipilimumab ± nivolumab, nivolumab, nivolumab ± vaccine, pembrolizumab	Baseline protein signature of 209 proteins discovered by use of MALDI–TOF and computational algorithms correlated with OS	(63)

CTC count	*n* = 7 (ICI) *n* = 42 (other treatments)	Ipilimumab, chemotherapy, targeted therapy	CTC count correlated with OS	

CTC (droplet digital PCR)	*n* = 49	Ipilimumab, nivolumab, pembrolizumab	Decrease in CTC within the first 7 weeks of ICI was linked to longer PFS and OS	(64)

Plasma ct-DNA: BRAF V600E/K, NRAS Q61K/R	*n* = 19 (ICI) *n* = 29 (targeted therapy)	Ipilimumab, nivolumab, pembrolizumab, targeted therapy	Pre-treatment ctDNA <10 copies/ml associated with response and longer PFS, decrease in ctDNA levels in responders of targeted therapy but not immunotherapy	(65)

**Stool Biomarkers**				

Gut microbiome	*n* = 26	Ipilimumab	Faecalibacterium and other Firmucutes associated with improved response, higher representation of Bacteroidetes related to poor response	(66)

	*n* = 89	PD1ab, not specified	Enrichment of Ruminococcaceae and Clostridiales found in responders, Bacteriodales in non-responders; Bacteroidales associated with shorter PFS	(67)

	*n* = 39	Ipilimumab + nivolumab, pembrolizumab	Enrichment of *Bacteroides caccae* in responders	(68)

	*n* = 39	PD1ab, not specified	Relative abundance of *Bifidobacterium longum, Collinsella aerofaciens*, and *Enterococcus faecium* in responders	(69)

*CRP, C-reactive protein; CTLA4ab, anti-CTLA-4 antibody; LDH, lactate dehydrogenase; MALDI–TOF, matrix-assisted laser desorption/ionization–time-of-flight; mo-MDSCs, monocytic myeloid-derived suppressor cells; NLR, neutrophil-to-lymphocyte ratio; PD1ab, anti-PD-1 antibody; PFS, progression-free survival; OS, overall survival; TNF-α, tumor necrosis factor-alpha; CTC, circulating tumor cells; ctDNA, circulating tumor DNA; sCTLA4, soluble CTLA4; sPD-L1, soluble PD-L1; sCD25, soluble CD25*.

### Clinical Condition (Performance Status)

A good clinical condition expressed by the ECOG (Eastern Cooperative Oncology Group) performance status (ECOG PS 0) is associated with prolonged OS for patients receiving PD1ab treatment, as well as for patients receiving other melanoma treatments such as BRAF inhibitors (30, 70). For other entities such as NSCLC (71), association between performance status and OS is also well known. Because of its prognostic character, performance status is frequently used as a biomarker in enrichment designs of clinical trials, i.e., only biomarker-positive patients are included, in this case only patients with good performance status. Because patients with poorer performance are not included and are therefore unavailable for further analysis, enrichment design prevents information from being gathered on the prognostic versus predictive value of performance status (20). Significance of other patient characteristics for ICI response, such as sex and age, has only been found in single studies with PD1ab for melanoma (24).

### Immune-Related Adverse Events

Immune-Related Adverse Events are side effects caused by the activated immune system and are, therefore, a possible sign of successful immune checkpoint blockage. Several retrospective analyses have reported an association between CTLA4ab-induced irAE and a more favorable clinical outcome (Table 1). From a cohort of 86 patients, occurrence of irAE ≥ grade 2 CTC-AE was associated with improved response, PFS, and OS (31). In contrast, other studies focusing on irAE of any grade could not find this association for large ipilimumab-treated cohorts (37, 38). The development of autoimmune hypophysitis was found to be associated with prolonged OS (33). It has been observed in retrospective analyses of several groups receiving PD1ab therapy that incidence of irAEs is associated with a more favorable outcome. In a large cohort of 576 PD1ab-treated patients, response but not PFS was associated with irAE manifestation of any grade (32). For certain adverse events, vitiligo was found to be associated with response (34) and, similar to exanthema, with longer OS (35). Arthralgia of any grade was associated with response and significantly longer PFS (36). Here, median onset of arthralgia was 100 days after start of treatment and was caused by either arthritis or reactivated osteoarthritis in pre-damaged joints. It is worth noting that all these analyses were performed retrospectively and that there is a risk of guarantee-time bias, i.e., patients with early progression are less likely to develop irAEs because of a shorter treatment period. This bias can be controlled to some degree by use of landmark analysis, which was used in the above-mentioned reports.

## Blood Biomarkers

Blood-based biomarkers have several preferential characteristics and are, therefore, the focus of biomarker research. First, they are easily accessible, which enables analysis at several time-points. Second, they might be independent from intra and inter-tumor heterogeneity. Third, they might reflect multiple sites of interest, e.g., tumor cells, tumor microenvironment, and the patient’s immune system.

### Serum Biomarkers Correlating With Tumor Load

Lactate dehydrogenase (LDH) is a house-keeping enzyme which is released by rapidly growing tumors. Serum LDH therefore correlates with tumor burden. For melanoma, the prognostic significance of this biomarker is expressed by its inclusion in the American Joint Committee on Cancer classification (72). Serum LDH levels correlate with patients’ OS in various treatment regimens (73), including ICI (Table 1) (23, 30, 31, 39–45). Nearly all studies have found no correlation between baseline LDH and response. Only a dynamic change in LDH from baseline to week 12 was found to be associated with response (31, 45). Hence, despite the prognostic value of LDH, patients with elevated serum LDH can respond to ICI; LDH elevation does not, therefore, lead to an exclusion of patients from ICI treatment. Patients with very high levels of more than twice the upper limit of normal, however, did not benefit from either CTLA-4ab or PD1ab monotherapy (23, 31, 39). Importantly, even though targeted treatments by use of BRAF/MEK inhibitors are known to lead to fast tumor responses in highly advanced patients with BRAF-V600-mutated melanoma, patients with normal LDH still achieve the better clinical outcome from treatment (74). Hence, the best treatment sequencing of targeted and immunotherapy for patients with BRAF-mutated melanoma and normal LDH is not clear.

Another serum biomarker which correlates with tumor burden is the acute-phase protein C-reactive protein (CRP). It is a prognostic marker for melanoma, and elevated concentrations are linked to worse PFS and OS (75). CRP also significantly affects prognosis for other malignancies such as renal, gastrointestinal, lung, pancreas, hepatocellular, and bladder cancer (76). For ICI, only retrospective analyses are available. A normal CRP level at the start of treatment was associated with longer OS for ipilimumab-treated melanoma patients (43). Decreasing CRP levels from baseline to week 12 of CTLA4ab therapy were, as for LDH, associated with longer PFS and OS (45). In multivariate analysis of a small study of PD1ab-treated melanoma patients, baseline CRP levels were no independent biomarkers for OS (30). For PD1ab treatment of NSCLC, in contrast, elevated baseline CRP levels were shown to be associated with shorter PFS (77).

### Differential Blood Count Biomarkers

Immune checkpoint inhibition works *via* activation of T lymphocytes. Hence, the number of lymphocytes and other immune cells circulating might affect its efficacy. Several retrospective analyses have focused on this question. The role of neutrophils, which can display heterogeneous phenotypes and diverse functionality, is also important (78). Increased levels of neutrophils have been found in the peripheral blood of cancer patients; they might possibly be induced by cytokines such as granulocyte-colony stimulating factor (G-CSF), although no definite cause for neutrophilia in malignancies has been clearly shown (78). Pretreatment elevation of neutrophil count has been found to correlate with worse OS in ICI treatment of melanoma (Table 1). Increasing lymphocyte counts, in contrast, correlated with prolonged OS in ICI-treated patients (Table 1). The neutrophil-to-lymphocyte ratio (NLR) has been more frequently reported to be of prognostic, and potentially predictive, value by several authors using various cutoffs (NLR > 2–5). For ipilimumab-treated melanoma patients, high baseline NLR was associated with shorter PFS and OS (46, 48). For PD1ab treatment, high baseline NLR was linked to non-response (50) and to worse OS for melanoma (49, 79) and for several other types of cancer being investigated in phase I studies with PD1ab/PD-L1ab treatment (79). For example, NLR was associated with lower incidence of response, poor PFS, and OS for NSCLC (71, 80) and for RCC (81). It is worth noting that an association was also found between NLR and prognosis for melanoma patients treated by use of BRAF inhibitors (82). Overall, NLR certainly has prognostic value but is probably not treatment specific and no predictive ability has been observed so far. It has, however, also been shown that eosinophils correlate with clinical outcome in ICI treatment of melanoma. A high pre-ICI absolute or relative eosinophil count was associated with prolonged OS (23, 41). Dynamically, for melanoma patients treated by use of ipilimumab, eosinophil counts that increased with treatment correlated with response to ipilimumab (51).

Myeloid-derived suppressor cells (MDSCs) are important in melanoma and other malignancies. MDSCs have immunosuppressive potential, particularly by inhibiting activated T cells, and can be divided into two subgroups: granulocytic and monocytic myeloid-derived suppressor cell (mo-MDSC) (78). The number of mo-MDSC in the peripheral blood in particular has been correlated with prognosis for melanoma patients (51). In CTLA4ab treatment, the number of mo-MDSC has been found to negatively affect incidence of response and survival (41, 47, 51). In addition, mo-MDSC was negatively correlated with OS for CTLA4ab-pretreated melanoma patients receiving PD1ab (52) (Table 1). The development of cytometry by time-of-flight (CyTOF) has enabled in-depth analysis of peripheral blood immune cells. CyTOF can measure up to 50 proteins per cell. Use of CyTOF for ICI patients has shown that high incidence of classical monocytes (CD14^+^CD16^−^HLA-DR^hi^) are associated with response and improved PFS in PD1ab therapy for melanoma (53).

It is worth mentioning that all these potential markers have been found by retrospective exploratory analyses. They potentially have prognostic features but their predictive potential remains unclear. Furthermore, the above-mentioned publications used several different cutoffs. Prospective studies are needed to investigate a possible predictive value of these biomarkers.

### Biomarkers on Peripheral T Cells

T cells are the effector cells of ICI treatment. Therefore, in addition to the pure cell number of several subsets of T cells in the peripheral blood, a more detailed analysis might be beneficial. Retrospective examination of peripheral blood T cell subsets in ipilimumab-treated melanoma patients revealed that higher pre-treatment CD4^+^/CD25^+^/FoxP3^+^ Tregs was associated with favorable survival (41, 45) (Table 1). Tregs express high levels of CTLA-4 and might, therefore, be one of the main targets of ipilimumab. It was shown that more melanoma-reactive CD8^+^ cytotoxic T cells in the peripheral blood were detected in patients after treatment than before treatment (83). Preexisting immune responses were only infrequently boosted.

Most studies have focused on PD-L1 expression on tumor cells and macrophages in the tumor microenvironment, whereas PD-L1-expression on peripheral T cells has been studied to a lesser extent. High PD-L1 expression on peripheral T cells (CD4^+^ and CD8^+^) has been shown to be associated with worse PFS and OS for CTLA4ab treatment of melanoma (55). For an NSCLC cohort treated mainly by chemotherapy, high PD-1/PD-L1/PD-L2 expression on peripheral blood T cells was associated with shorter OS (84). PD-L1 expression on peripheral T cells might, therefore, be a mechanism for tumor immune escape. Expression of co-stimulatory molecules on peripheral T cells was also studied. Detectable levels of CD137^+^CD8^+^ cytotoxic T cells in the peripheral blood were found in patients with relapse-free status after adjuvant combined ICI, but this was not investigated in the therapeutic setting (55). CyTOF analysis revealed that high pre-treatment incidence of memory T cells was a potential marker for response to CTLA4ab, whereas higher incidence of distinct NK cell subsets was found to be associated with response to PD1ab treatment for melanoma (54).

Tumor antigen presentation by human leukocyte antigen class I (HLA-I) molecules is a prerequisite for cancer cell attack by cytotoxic T cells. Maximum heterozygosity at HLA-I loci (A, B, C) opposed to homozygosity for at least one HLA locus was shown to be associated with longer OS after ICI for mainly NSCLC and melanoma (56). Furthermore, HLA-B44 supertype was linked to prolonged OS, whereas the HLA-B62 supertype or somatic loss of heterozygosity at HLA-I were associated with worse OS for melanoma (56). Although assessed in test and validation cohorts, these biomarkers have also not been prospectively tested for their potential predictive versus prognostic value. Nevertheless, this investigation indicates that diversity in antigen presentation might improve tumor defense. Tumor cell antigens presented on MHC molecules are recognized by T cells *via* the T cell receptor (TCR). The TCR is, therefore, of great interest in ICI. TCR diversity and clonality can be investigated by use of sequencing methods. Most investigations focus on TCR sequencing in tumor tissue specimens (see “Tissue Biomarkers”). TCR sequencing data in the peripheral blood are limited. In ipilimumab-treated melanoma patients, patients with a positive clinical outcome had a higher degree of TCR repertoire richness prior to therapy (57). In patients with urothelial carcinoma, TCR sequencing in peripheral blood was done before and after atezolizumab administration. Here, a pretreatment TCR clonality below the median was associated with improved PFS and OS (85). Furthermore, a long-lasting clinical benefit was found in patients with a more substantial expansion of tumor-associated TCR clones after three weeks (85). T cells carrying the γδ-TCR-subtype—as opposed to the more common αβ-subtype—play a distinct role in anti-tumor immunology. A study found that higher incidence of Vδ2^+^ cells (versus Vδ1^+^ cells) was linked to longer OS in melanoma patients, and suggested that Vδ2^+^ cells potentially have tumor-killing capability (86). However, this has not yet been investigated for ICI.

In summary, T cells as the effector cells of ICI are the focus of biomarker research for melanoma and other malignancies. Some approaches are promising, but no biomarker has yet been evaluated in a prospective clinical setting. Their predictive ability therefore remains to be determined.

### Soluble Serum Biomarkers

Soluble serum biomarkers that might correlate with clinical benefit of ICI treatment include immune regulatory molecules such as cytokines or soluble checkpoint receptors and binding partners. Biomarker potential in ICI treatment of melanoma (Table 1) and other malignancies has been found for several soluble serum factors.

Soluble CTLA-4 (sCTLA4), which is mainly secreted by regulatory T cells (Tregs), has inhibitory effects on T cell immune responses (87). An association has been found between higher sCTLA4 levels and both response and prolonged OS for a small cohort of ipilimumab-treated melanoma patients; this was not found for patients who did not receive ipilimumab (58). In view of its inhibitory function on T cells, neutralization by CTLA4ab therapy might be responsible for this finding. Higher levels of soluble PD-L1 (sPD-L1) can be found in tumor patients compared with healthy individuals (88, 89). Its physiological role has not yet been identified (89). It holds some prognostic value because high pre-treatment concentrations are associated with shorter OS for NSCLC (88), and for hepatocellular carcinoma (90), and it is linked to disease progression in ICI for melanoma (59). sPD-L1 is, however, likely to be only prognostic and not predictive for melanoma, because assessments pre- and during early ICI did not reveal significant associations with response or OS (59). In addition to sPD-L1, a soluble form of PD-1 (sPD-1) also exists (89) which is currently being investigated in a clinical trial (NCT03197636).

Soluble ligands of the transmembrane receptor NKG2D (sULBP-1, sULBP-2), which affect induction or reactivation of T cell responses, were associated with OS for ICI-treated but not for BRAF inhibitor-treated melanoma patients (44). They are interesting biomarkers with treatment-specific potential. Further data are, however, needed to confirm the significance of these markers. In addition, soluble CD25 (sCD25), the alpha unit of the IL-2 receptor, was found to be a biomarker in ipilimumab therapy. The interleukin (IL)-2/IL-2 receptor pathway is essential for the antitumor activity of CTLA4ab (91). High pre-treatment serum levels of sCD25 were shown to be associated with shorter OS for CTLA4ab-treated patients (60). A possible explanation for this finding could be direct binding of sCD25 to IL-2, which would amplify Tregs and inhibit tumor immune response.

It has been shown that other serum factors including vascular epithelial growth factor and chemokines such as C-X-C chemokine motif ligand (CXCL)8 are of prognostic significance for PFS and OS of melanoma of different stages, regardless of treatment (92). For CTLA4ab therapy, elevated pre-treatment levels of CXCL11 were associated with poor OS (61). The gene expression of CXCL11 is induced by interferon-(IFN)-γ, and CXCL11 binds to its chemokine receptor CXCR3, which is mainly expressed on activated T cells. CXCR3 is highly important for the migration of cytotoxic T cells, and its tissue expression correlates with poorer prognosis for several malignancies (93). Elevated CXCL11 in blood has been linked to poorer outcome for ipilimumab-treated melanoma (61). For PD1ab treatment, serum IFN-γ, IL-6 and IL-10 levels were significantly higher for responders than for non-responders (62).

In contrast to single-biomarker searches, serum-based multi-marker assays are of current and future interest. One group developed a test based on 119 patients with pre-PD1ab therapy for metastatic melanoma using matrix-assisted laser desorption/ionization-time of flight (MALDI–TOF) mass spectrometry, which was validated in four independent cohorts (63). Computational algorithms were used for data analysis, resulting in a protein signature of 209 proteins that appears to differentiate patients with three-year OS of over 50% from patients with three-year OS of less than 20%. Further analysis revealed that acute phase proteins, complement, and wound healing pathways were associated with poor outcome (63). Because this test has also not been prospectively evaluated yet, distinction of prognostic versus predictive ability is warranted.

### Liquid Biopsy

Liquid biopsy, including CTCs, ctDNA, and circulating tumor RNA (ctRNA) has only been studied in small patient cohorts treated by use of ICI (mainly CTLA4ab) for melanoma. An association between treatment response and a decrease in CTCs and ctDNA has been found for targeted therapy, but not for ICI (94). Methodological improvements offer new opportunities for CTC detection, as has been very recently described for microfluidic enrichment of melanoma CTCs combined with RNA-based droplet digital PCR quantitation (64). That study found that a decrease in CTCs within the first seven weeks of ICI was linked to prolonged PFS and OS in CTLA4ab or PD1ab-treated melanoma patients (64). Another approach to CTCs is characterization of subsets of CTCs that express specific markers. The heterogeneity of melanoma CTCs and the significance of CTC subsets (e.g., receptor activator of NF-κβ (RANK) expressing CTCs) as biomarkers has been found to affect targeted treatment; this was not, however, observed for ICI (65). For NSCLC, high expression of carcinoembryonic antigen and telomerase reverse transcriptase was linked to non-response to nivolumab (95). For urothelial carcinoma, CTCs with high PD-L1 expression were associated with worse OS (96), and for chemotherapeutically treated SCCHN, high levels of CTCs with PD-L1 expression were linked to poor PFS and OS (97). CTCs are being studied as part of a recruiting clinical trial on prospective biomarkers for melanoma, and this will hopefully shed light on a potential predictive function of CTCs for ICI.

Not only CTCs but also ctDNA has been investigated in PD1ab therapy. A proof-of-concept study found that detectable levels of ctDNA in week 8 of PD1ab therapy were linked to worse PFS and OS for NSCLC, uveal melanoma, and microsatellite-instable colorectal cancer (98). Furthermore, an association was found between high hypermutated ctDNA levels and response, PFS, and OS for diverse malignancies treated by use of ICI (99).

### Other Blood Biomarkers

Several other blood biomarkers have been investigated in ICI patients; for example, blood-based testing of gene-expression profiles of cathepsin D, phopholipase A2 group VII, thioredoxin reductase 1, and interleukin 1 receptor-associated kinase 3 were found to be associated with OS for CTLA4ab-treated patients (100). Ongoing studies on blood biomarkers for ICI treatment of melanoma include assessment of different T cell subsets, cytokines, and CTCs (Table 1). The challenge is to select the most promising biomarkers, ideally identified by several different investigators, and to study them in prospective clinical trials.

## Stool Biomarkers

The effect of gut microbiota on anti-tumor response has recently been observed in both murine and human studies for several cancers, including melanoma, NSCLC, and RCC. Unsurprisingly, microorganisms are prevalent in primary CRC, but distant metastases are also colonized with Fusobacterium and its associated microbiome, including Bacteroides, Selenomonas, and Prevotella species (101). Gut flora composition can stimulate or inhibit immune response. Immunostimulatory effects of Bacteroidales, particularly *Bacteroides fragilis*, have been observed for CTLA4ab therapy in mice (102). Similarly, Bifidobacterium improved anti-tumor responses for PD-1/PD-L1 blockade in a murine melanoma model (103). In contrast, ICI treatment itself can affect the population of gut microbiota (102). Baseline gut microbiota have been investigated in small CTLA4ab-treated melanoma cohorts (Table 1). Enrichment with Faecalibacterium and other Firmucutes was associated with improved response and with development of colitis, whereas a higher representation of Bacteroidetes was related to poorer response to CTLA4ab therapy (66). For melanoma patients receiving PD1ab therapy, enrichment of Ruminococcaceae and Clostridiales was found in responders whereas Bacteriodales were enriched in non-responders (67). Shortened PFS was observed for patients with high abundance of Bacteroidales, which is in agreement with another publication on melanoma patients treated with CTLA4ab (66). Another analysis found enrichment of *Bacteroides caccae* in all ICI responders, and specifically *Faecalibacterium prausnitzii, Bacteroides thetaiotaomicron*, and *Holdemania filiformis* if treated with ipilimumab plus nivolumab combination therapy. *Dorea formicogenerans* was enriched in pembrolizumab responders (68). Other authors found relative abundance of *Bifidobacterium longum, Collinsella aerofaciens*, and *Enterococcus faecium* in PD1ab responders with melanoma (69). An imbalance in gut microbiota correlating with impaired immune cell activity was observed for non-responders. Treatment by use of antibiotics before or shortly after ICI was associated with poorer response and worse OS for patients with RCC and NSCLC; in this analysis, a higher percentage of non-responders (69%) had a particularly low level of *Akkermansia muciniphila* compared with responders (34%) (104). It is worth nothing that in a mouse model, fecal transplants of responders into germ-free mice restored the anti-tumor effect of PD-1/PD-L1 blockade (67, 69, 104).

In summary, gut microbiota affect anti-tumor immune response. However, there is only partial overlap between the potentially relevant microorganisms (Table 1). It is unclear if this is because of methodological reasons, or if it depends on the individual tumor entity, or the geographical region and associated dietary habits of the investigated patients. Further prospective studies are needed to evaluate the prognostic or predictive effect of gut microbiota on ICI outcome (currently ongoing: NCT02960282, NCT03370861). A study on fecal microbiota transplantation for metastatic melanoma patients who failed ICI is also being conducted (NCT03353402).

## Tissue Biomarkers

### PD-L1 Expression

In the initial phase I study of nivolumab for patients with solid tumors, an association between PD-L1 expression and probability of response was observed for NSCLC, melanoma, and RCC (105, 106) (Table 2). Because clinical significance was greatest for NSCLC, this led to further PD1ab studies using enrichment designs with different antibodies and expression cutoffs (107–109). Because patients with PD-L1 negative (or PD-L1 expression below cutoff) value cannot be followed in clinical trials of enriched design, it is not possible to distinguish between prognostic and predictive value (20). It is worth mentioning that in a retrospective analysis of metastatic melanoma patients, PD-L1 expression was linked to improved OS irrespective of treatment type; this raises the possibility of a prognostic rather than a predictive value for ICI (28). Throughout several clinical trials for NSCLC and urothelial cancer, no association was observed between PD-L1 expression and ICI therapy outcome (8, 110, 111). There was one exception: for urothelial carcinoma, a composite biomarker of either ≥25% positive tumor cells or ≥25% positive immune cells indicated tumor response to durvalumab, and is a pre-requisite for treatment according to FDA-approval (15). PD-L1 positivity was also associated with higher probability of response in a subgroup analysis of PD-L1ab therapy for MCC (14). In addition, PD-L1 expression on tumor and immune cells was linked to higher incidence of response for SCCHN (112). There is growing evidence that response is associated more with PD-L1 expression on tumor-infiltrating immune cells than it is with tumor cell PD-L1 expression (113).

**Table 2:** Tissue and imaging biomarkers for clinical outcome under checkpoint blockage for metastatic melanoma patients.

Biomarker	Number of patients	Treatment	Results	Reference
**Tissue Biomarkers**				

PD-L1 expression	*n* = 41–43 (melanoma, NSCLC, renal cell carcinoma, and others)	Nivolumab, atezolizumab	PD-L1 expression on tumor or TILs associated with response	(105) (106) (113)

	*n* = 945 (stratified for PD-L1 expression)	Ipilimumab, nivolumab, ipilimumab plus nivolumab	Patients with PD-L1 negative tumors had longer PFS and OS under combined ICI compared to nivolumab monotherapy	(5) (114)

Tumor-infiltrating lymphocytes (TILs)	*n* = 82	Ipilimumab	High baseline FoxP3 and IDO expression and increase in TILs from week 0 to week 3 associated with disease control	(115)

	*n* = 20	Nivolumab, pembrolizumab	Partially exhausted (PD-1^high^CTLA-4^high^) tumor-infiltrating CD8^+^ T cells correlated with response and longer PFS	(116)

	*n* = 16–46	Pembrolizumab	Cytotoxic T cells at tumor margins associated with response, higher clonal expansion of TCR in responders	(117)

	*n* = 32–33	Nivolumab, pembrolizumab, atezolizumab	TCR clonality not associated with outcome	(118, 119)

Mutational load, neoantigen load	*n* = 38–110	Ipilimumab, tremelimumab, nivolumab, pembrolizumab	High mutational and neoantigen load associated with clinical benefit (response, DCR > 6 months, PFS, OS)	(85, 120) (121) (122) (123) (124)

	*n* = 68	Nivolumab	Mutational and neoantigen load decreased with treatment in responders	(125)

Single mutations	*n* = 229	IL-2, CTLA4ab, PD1ab, PD-L1ab, not specified	NRAS mutation correlated with disease control and longer PFS	(126)

	*n* = 32–33	Nivolumab, pembrolizumab, atezolizumab	NF-1 mutation associated with mutational load and response, NRAS-mutations not associated with clinical outcome	(118, 119)

	*n* = 38	Nivolumab, pembrolizumab	Tumors from responders were enriched for BRCA2 mutations	(122)

Histological subtype	*n* = 60 (desmoplastic melanoma)	Nivolumab or pembrolizumab ± ipilimumab, PD-L1ab, not specified	Desmoplastic melanoma showed higher response rates as reported in the literature (probably because of high mutational burden)	(127)

MHC-I/II expression	*n* = 23–30	Nivolumab, pembrolizumab, atezolizumab	MHC-II positivity on tumor cells associated with response, PFS, and OS	(118, 119)

Gene expression	*n* = 21–45	Ipilimumab	High IFN-γ expression and of IFN-γ-inducible genes (e.g., CXCL9, CXCL10, and CXCL11) correlated with longer PFS, OS	(128) (129)

	*n* = 43 (melanoma only)	Atezolizumab	Expression of baseline T helper type 1, CTLA4, and IFN-inducible genes (e.g., IDO1, CXCL9) as well as the absence of CX3CL1 associated with response	(113)

**Imaging Biomarkers**				

Tumor burden measured by CT (RECIST1.1)	*n* = 593	Pembrolizumab	Lower baseline tumor burden (RECIST 1.1) associated with longer OS	(26)

FDG-PET/CT	*n* = 22	Ipilimumab	FDG-PET/CT (EORTC criteria) at week 5 predicts disease progression while response could not be identified	(130)

	*n* = 20	Ipilimumab (*n* = 16), nivolumab (*n* = 1), PD-L1ab BMS-936559 (*n* = 3)	FDG-PET/CT at week 3–4 predicted best response at ≥4 months [using RECIST 1.1, immune-related response criteria, EORTC criteria, and PET response criteria in solid tumors (PERCIST)]	(131)

	*n* = 41	Ipilimumab	Cutoff of four newly emerged FDG-avid lesions on PET/CT after 12 weeks indicates treatment failure, SUV changes did not correlate with clinical outcome	(132)

FDG-PET/MRI (PERCIST)	*n* = 10	PD1ab, not specified	Metabolic response at week 2 might indicate response at 3 months	(133)

*CT, computerized tomography; CTLA4ab, anti-CTLA-4 antibody; FDG, ^18^F-fluoro-deoxy-glucose; MRI, magnetic resonance imaging; PET, positron emission tomography; PD1ab, anti-PD-1 antibody; PFS, progression-free survival; OS, overall survival; SUV, standard uptake value; DCR, disease control rate; TCR, T cell receptor*.

PD-L1 expression on tumor cells was used to stratify the design of the Checkmate-067 trial investigating the combination of ipilimumab plus nivolumab compared with nivolumab and ipilimumab monotherapies (5). Although not designed for this purpose, this study revealed that patients with tumors expressing PD-L1 had similar PFS and OS compared with PD1ab monotherapy and the combination of CTLA4ab plus PD1ab (5, 114). Response to combined ICI was still higher, however, compared with response to PD1ab monotherapy. The study was not designed to compare the two nivolumab-containing treatment arms, but it shows possible limitations of PD-L1 as a biomarker for treatment decisions for melanoma.

General problems associated with PD-L1 as a biomarker are: use of different immunohistochemical (IHC) assays, different cutoffs, intra-tumor heterogeneity, and dynamic changes of PD-L1 expression. In summary, there are conflicting data in diverse tumor entities. It is worth noting that treatment responses can be found in PD-L1-negative tumors.

### Tumor-Infiltrating Lymphocytes (TIL)

The presence of TILs has prognostic potential for different tumor entities regardless of tumor stage (134, 135). For patients with metastatic melanoma, TILs were associated with a better outcome for primary melanoma and metastatic disease, irrespective of treatment type (28, 136). A prospective biomarker study of ipilimumab-treated patients with melanoma found an association between early increase in TILs and disease control (115) (Table 2). A more detailed assessment of the T cell infiltrate at baseline revealed an association between high baseline FoxP3^+^ Tregs and indoleamine-2,3-dioxygenase (IDO) expression and favorable outcome (115). For PD1ab-treated melanoma patients, no association was found between baseline TILs and response to PD1ab (106). Abundance of partially exhausted (PD-1^high^CTLA-4^high^) tumor-infiltrating CD8^+^ T cells correlated with response and PFS in PD1ab therapy (116). Cytotoxic T cells at tumor margins were also linked to response to PD1abs (117). Preliminary investigations on TCR clonality of TILs in melanoma metastases revealed higher clonal expansion of TCR for PD1ab-responders compared with non-responders (117). This finding was not, however, confirmed by other authors (119).

Interferon-γ is one of the cytokines secreted by activated T cells and is known to upregulate PD-L1 expression. This might be one reason why PD-L1 expression could be co-localized with TIL infiltrates in melanoma metastases (117, 137). In a retrospective analysis, pretreatment tumor samples from NSCLC and melanoma patients treated with PD1ab were evaluated for IFN-γ expression (129). A significantly longer PFS and OS were observed for patients with high IFN-γ expression. High pre-treatment expression of IFN-inducible genes (e.g., IDO1, CXCL9, CXCL10, and CXCL11 among others) was associated with response and prolonged OS for PD-L1ab treatment of melanoma, but this was less pronounced for NSCLC or RCC (113, 128). Primary mutations in IFN-γ signaling pathways (e.g., JAK1 and JAK2 mutations) have been described for several tumor entities. For cutaneous melanomas, JAK1/2 mutations were detected before treatment in 21% of tissue specimens (138). Interestingly, patients with resistance to ICI were found to harbor JAK mutations with consecutive loss of IFN-γ pathways (139, 140).

The following challenges apply for all potential tissue biomarkers described above: possible dependency on biopsy site, the specific time of the biopsy, and intratumor-heterogeneity. It should be noted that, when considering multiple potential biomarkers, large multivariable analyses are required to exclude a significant overlap of markers (138). Moreover, future models might include transcriptome-derived stromal and immune cell scores exceeding a pure TIL assessment (141).

### Mutational Analysis

The first notion that mutational changes might affect tumor response came with the observation that melanoma patients with a high mutation rate benefitted more from ipilimumab treatment than patients with a low one, resulting in longer OS (120). In agreement with this, tumors with a naturally high mutation rate because of exogenous cancerogens, such as UV light, smoking, and alcohol (melanoma, lung cancer, SCCHN, and bladder cancer), belong to the entities that respond best to ICI treatment (123, 142–145). A small biomarker-stratified trial was performed for non-colorectal CRC and mismatch-repair deficient cancers. Stratification according to mismatch repair deficiency and mismatch-repair proficiency revealed a 40% response for patients with mismatch-repair deficiency (MSI high, dMMR), whereas mismatch-repair proficient patients did not respond at all (146). Whereas mismatch-repair deficiency can be found in gastrointestinal and genitourinary tumors (147, 148), it is of minor significance for melanoma. Colli et al. suggested a cutoff of 192 nonsynonymous mutations for a potential clinical benefit of ICI (149). Here, a high mutational load seems to result in prolonged OS in particular, whereas no correlation with response to PD1ab was observed (122). This is in agreement with the observation that melanoma patients treated with PD1ab survive longer even when not responding to the treatment (150). This was found to change, however, for ipilimumab treatment prior to PD1ab therapy; in contrast to ipilimumab-naïve patients, no association between tumor mutational burden and response/OS was observed (125). Most likely, the difference is not because of the number of mutations, but because of the increasing chance of tumor neoepitopes which might be easier recognized by the immune system. Clonal neoantigens in particular might significantly affect ICI outcome (120, 123).

A considerable difficulty of using mutational load as a biomarker is its practical implementation in clinical routine. In addition to the high costs incurred by whole exome sequencing, most neoantigens are probably patient-specific and not recurrent (121). Specific types of mutations might be more frequent, e.g., the frameshift insertion and deletion count was found to be associated with ICI response for melanoma (124). Furthermore, several groups have proposed fitness models to describe neoantigen qualities that could be possibly employed as biomarkers in the future (151, 152). It is not clear, however, that neoantigen burden will add significant value to mutational burden testing, as shown in the examples of urothelial carcinoma and melanoma (85, 153).

Tumor antigen presentation is essential for the immune defense of cancer, and mutations affecting pathways important for antigen presentation, e.g., beta-2-microglobuline (B2M) loss, might result in ICI resistance (139). B2M mutations are more frequent for melanoma, bladder, gastric, and lung cancer in particular, with 27–50% found for these cancers in The Cancer Genome Atlas dataset compared with 1.8% across all tumor types (138). The MHC-II-expression itself (HLA-DR^+^) was found to be associated with PD1ab/PD-L1ab response in melanoma (118).

It would be easy to use biomarkers for mutations that are routinely assessed in clinical care. BRAF-V600 mutations, which are found in 40–50% of melanomas, are not associated with ICI outcome (154–157). A subgroup survival analysis of combined ipilimumab plus nivolumab versus PD1ab monotherapy showed a trend toward longer OS for combined ICI treatment of BRAF-mutant patients, but this needs to be addressed further (114). In a retrospective analysis from the pre-ICI era, NRAS mutations, which are seen in up to 20% of melanomas, were found to be associated with worse OS (158). After introduction of ICI, a retrospective study investigating patients treated by immunotherapy, including IL-2, CTLA4ab, PD1ab, and PD-L1ab revealed greater disease control and longer PFS for NRAS-mutant melanoma (126). This result was not, however, found for a smaller cohort of patients with PD1ab/PD-L1ab therapy (119). The NF-1 mutation, which is associated with UV damage and high mutational load, was linked to higher incidence of response and prolonged survival for PD1ab-treated patients (119). A more favorable response to PD1ab therapy was observed for desmoplastic melanomas which are characterized by a high mutational load and frequent NF-1 mutations (127); it should be mentioned that this observation was also made from retrospective assessment. For NSCLC, single-gene mutation analysis showed that the presence of an EGFR-mutation seems to be a negative predictor for PD1ab response (159). NGS data, however, revealed a lower mutational burden for EGFR-mutant NSCLC, which could be one reason for this finding (160).

Objectives for the future include exploration and validation of a panel of genetic biomarkers detected by next-generation sequencing. Definition of cutoffs is a current challenge because absolute values depend on the depth of sequencing. Furthermore, gene translocations/fusions and other variants will not be detected by use of targeted sequencing techniques. Development of multi-marker assays is more complex and specific computational algorithms must be used for validation (20).

## Imaging Biomarkers

### Anatomic Imaging

The use of imaging enables non-invasive assessment of tumor dimensions and can also provide biologic tumor data. The current standard assessment procedure for metastatic melanoma and other advanced malignancies is computerized tomography (CT) with iodine contrast dye, evaluated according to response evaluation criteria in solid tumors (RECIST) 1.1 (161). It has been shown that the size of baseline tumor lesions is associated with OS (26, 162). This is probably of prognostic value because it correlates with tumor load. However, RECIST 1.1 might be insufficient to evaluate response to ICI therapy, in particular cases of initial tumor progression or occurrence of new lesions during ICI. To overcome this problem, immune-related response criteria (irRC and irRECIST) have been introduced as alternative response criteria (163). Pure anatomic imaging is, however, unlikely to be sufficient to predict tumor response to ICI. New imaging biomarkers for metabolic and immune imaging are discussed below.

### Metabolic Imaging

In addition to anatomic imaging, metabolic imaging by use of ^18^F-fluoro-deoxy-glucose-positron emission tomography (FDG-PET) can add clinically meaningful data when imaging malignancies. Two different response criteria for FDG-PET imaging are currently used in clinical routine: European Organisation for Research and Treatment of Cancer (EORTC) criteria and positron emission tomography response criteria in solid tumors (PERCIST) (164, 165). It has been shown that FDG-PET combined with CT is of clinical value for assessment of ICI responses in melanoma (130–132) (Table 2). The assumption for metabolic imaging is that metabolic changes in tumors occur prior to anatomic changes, which might enable prediction of response to ICI earlier during the course of treatment. This can be prevented by the failure to discriminate between inflammation and tumor metabolism (166). For ipilimumab, one clinical trial showed that FDG-PET/CT 5 weeks after treatment initiation could predict disease progression to CTLA4ab; patients responding to treatment could not be identified at this time (130). In another trial it was possible to predict best response by use of PERCIST and EORTC criteria (131). To evaluate ICI response, a new PET-CT classification, the PET response evaluation criteria for immunotherapy criteria, was developed to reflect the fact that single new lesions do not define disease progression. The absolute number of new lesions was, however, more important than changes in standardized uptake values (SUV) (132). During PD1ab therapy for melanoma, use of FDG-PET/magnetic resonance imaging (MRI) as early as 2 weeks after the start of treatment might identify patients with complete response at the 3-month time-point (133). For FDG-PET/CT for NSCLC, maximum SUV at 4 weeks after commencement of PD1ab therapy was associated with PFS and OS (167). However, these are case series and small prospective studies. Larger prospective trials are needed to investigate these findings further.

^18^F-fluorothymidine-PET (FLT-PET) uses a thymidine analog that accumulates in proliferating tissues, including malignant and immune cells (168). Positive findings were published for e.g., differentiation of cerebral radionecrosis from glioma progression (169), and correlation of mean SUV with OS for resectable pancreatic carcinoma (170). In contrast, no association was found between early changes in FLT uptake after the first cycle of chemotherapy for CRC and the response evaluated from subsequent CT scans (171). Its value as a biomarker for ICI in melanoma has only been reported in one case of FLT-PET/MRI; this precludes general conclusions (172). Further clinical evaluation of FLT-PET in ICI is, therefore, warranted. O-(2-[18F]fluoroethyl)-l-tyrosine (FET-)PET can be used to specifically image primary brain tumors and metastases to the brain which can be differentiated from healthy, inflamed, and radionecrotic tissue (173, 174). FET-uptake correlates with ki-67 expression and could be a potential biomarker for early response assessment (175). For melanoma, a case report showed that pseudo-progression of melanoma brain metastases could be detected by use of FET-PET (176) but more data are needed to assess the value of FET-PET in ICI.

Modern MRI techniques, including dynamic contrast-enhanced MRI, are also available for high resolution imaging of tumor perfusion or cell-membrane permeability (177). This technique could be particularly useful for assessment of specific metastatic sites, e.g., hepatic metastasis (178). Another potential application could be MRI-based immune-cell tracking and assessment of drug delivery (179).

With the exception of FDG-PET/CT, all the methods described above have been studied in only a few patients, and rarely in the setting of ICI. Results of ongoing studies will reveal a potential prognostic or predictive value of PET-biomarkers.

### Immuno-Imaging

Several immuno-PET tracers, namely monoclonal antibodies, scaffold proteins, or peptides have been evaluated in preclinical tumor models. Potential targets are, for example, CD8^+^ cytotoxic T cells, PD-1, and PD-L1. Immuno-PET tracers have been studied in preclinical models. A ^89^Zr-labeled PEGylated single-domain anti-CD8 antibody was used for longitudinal evaluation of CTLA4ab treatment in the B16-melanoma mouse model. A homogeneous distribution of the anti-CD8 PET signal was observed for responding animals, whereas a heterogeneous signal was associated with lower response and faster tumor growth (180). Another group studied a ^89^Zr-desferrioxamine-labeled anti-CD8 cys-diabody in PD1ab treatment of Balb/c mice with CRC. They found a higher SUV in responding animals compared with non-responding ones. They also found that uptake for responders tended to be intra-tumoral, whereas uptake for non-responders was in the margins of the tumor (181); this is in agreement with the role of intra-tumoral CD8^+^ cytotoxic T cells in PD1ab response.

Another T cell imaging approach is *via* visualization of PD-1. The feasibility of this method has been proven in murine studies. Natarajan et al. developed the anti-PD-1 tracers ^89^Zr-keytruda and ^64^Cu-keytruda; these were evaluated in a humanized NOD-scid mouse model, and uptake in tumors and lymphoid tissue was observed for human melanoma tumors (182). Other groups developed radiotracers which target PD-L1 expressed on tumor cells and on immune cells of the tumor microenvironment (183–185). The feasibility of PD-L1 imaging was shown by use of ^64^Cu-atezolizumab in mice with tumors constitutively expressing PD-L1, and in two breast cancer mouse models (183). Investigation of irradiated versus non-irradiated tumors in a HPV + SCHNN and a B16F10 melanoma mouse models by use of an ^89^Zr-labeled anti-PD-L1 monoclonal antibody revealed PD-L1 upregulation in irradiated tumors specifically (184). In a patient-derived xenograft model of NSCLC, the ^89^Zr-C4-PD-L1 antibody revealed PD-L1 changes after chemotherapy (185).

These immuno-PET tracers have been investigated in animal models, which can certainly improve understanding of response or non-response mechanisms to ICI treatment. Studies in humans are under way. A possible predictive ability of immuno-PET in the setting of ICI, however, needs to be explored in the future (NCT03313323, NCT02760225).

## Conclusion

Several factors might affect response to ICI treatment, including mutational load, tumor microenvironment, and stool microbiome. Upfront exclusion of metastatic melanoma patients from ICI therapy on the basis of biomarkers is not currently possible. It also remains unclear which patients will need combined ICI and which patients will benefit from use of PD1ab only. Although there are several potential biomarkers, their predictive versus prognostic abilities have not yet been validated by prospective clinical trials. In particular, the best sequence of treatment to follow, e.g., targeted versus immunotherapy for melanoma, cannot be answered on the basis of the biomarker data currently available.

Peripheral blood immune-cell analysis, e.g., by use of CyTOF, enables investigation of multiple markers, and will hopefully reveal predictive biomarkers in future. A multi-marker assay is more likely than a single biomarker. Future challenges include the development and validation of multi-marker assays, which will require detailed pre-analytics, computation algorithms, and, most importantly, well-designed clinical trials with large numbers of patients.

## Author Contributions

KB-B and JH contributed to the conception and design of the article, acquisition and interpretation of data, drafting of the article, critical revision, and final approval. Both are accountable for the content of this manuscript.

## Conflict of Interest Statement

KB-B has received honoraria and travel reimbursements from MSD Sharp & Dome GmbH Oncology (MSD) and Roche Pharma AG (Roche). JH has received consultancy fees from Amgen GmbH and MSD, payment for lectures from BMS, MSD, Roche, GSK, Novartis, and Pfizer, and travel reimbursements from BMS, MSD, Amgen, and GSK.
